# Composition of Necrophagous Insect Assemblages in Altitudinal Gradient of Central Chile

**DOI:** 10.3390/insects17010019

**Published:** 2025-12-23

**Authors:** Melissa Marzolo Bown, Patricia J. Thyssen, Aline Marrara Prado, Cristian Villagra

**Affiliations:** 1 Laboratorio de Ecología Sensorial, Instituto de Entomología, Facultad de Ciencias Básicas, Universidad Metropolitana de Ciencias de la Educación, Santiago 7760197, Chile; 2 Facultad de Ciencias, Universidad de Chile, Santiago 7800003, Chile; 3 Laboratory of Integrative Entomology, Department of Animal Biology, Institute of Biology, Universidade Estadual de Campinas, São Paulo 13083-862, Brazil; pthyssen@unicamp.br (P.J.T.); alinemarrara123@gmail.com (A.M.P.)

**Keywords:** detritivorous insects, bioindicators, eco-forensics, altitudinal environments

## Abstract

This study evaluated whether necrophagous arthropod assemblages, with emphasis on Calliphoridae (Diptera), differ among three sites in central Chile: Coastal (50 masl), Pre-mountain range (1000 masl), and High Andean (1800 masl). We analyzed differences in the abundance, richness, and community composition of arthropods associated with decomposed bait. Specimens were collected using carrion traps and active sampling. In total, 1240 specimens (predominantly insects) were obtained across three orders: Diptera (n = 1216), Hymenoptera (n = 22), and Neuroptera (n = 1). Within Diptera, the families recorded were Calliphoridae (n = 947), Muscidae (n = 118), Piophilidae (n = 77), Fanniidae (n = 44), Sarcophagidae (n = 15), Mycetophilidae (n = 8), Phoridae (n = 5), and Ulidiidae (n = 2). No significant differences were detected in total arthropod abundance or Calliphoridae abundance among sites. Necrophagous arthropod richness was highest at the Coastal and Pre-Andean sites. For Calliphoridae, no significant differences were observed among sites for the evaluated ecological parameters. Indicator taxa at the Coastal site included Braconidae (Hymenoptera) and the dipteran families Fanniidae and Muscidae. At the High Andean site, blowflies (Diptera: Calliphoridae), including *Chrysomya albiceps*, *Compsomyiops fulvicrura*, *Lucilia cuprina*, and *Lucilia sericata*, were identified as indicators. All Calliphoridae species sampled were confirmed as potentially forensically relevant for the region due to their necrophagous habits. Beyond documenting Calliphoridae across elevation, this study provides baseline data to support future work using this taxon to assess environmental impacts, characterize local trophic interactions, and inform forensic applications.

## Simple Summary

Necrophagous insects are key organisms for studies spanning environmental monitoring and applied contexts. Here, we tested whether abundance, richness, and community composition of necrophagous arthropod assemblages differ among three sites along an altitudinal gradient (50, 1000, and 1800 masl) in the Andean cline of Central Chile. Specimens were collected by active sampling and carrion trapping. Arthropod taxon richness differed markedly among sites, with the highest diversity at low and intermediate elevations. At 1800 masl, blowflies (Diptera: Calliphoridae), including *Chrysomya albiceps*, *Compsomyiops fulvicrura*, *Lucilia cuprina*, and *Lucilia sericata*, were identified as indicator species. The presence of Calliphoridae at high elevation underscores the value of ecology-focused studies of necrophagous insect diversity and community composition for environmental monitoring and supports the use of these data to inform future research and forensic applications across altitudinal gradients.

## 1. Introduction

As abiotic and biotic conditions change sharply along elevational gradients, elevational clines have been proposed as natural laboratories for studying how organisms respond to spatial variation in environmental stressors [1,2]. This variation has also been used as a proxy to evaluate shifts in species distributions expected under climate-driven changes in environmental variables [3,4]. Species occurring at different elevations often exhibit distinct physiological responses to these ecological factors, which in turn shape their distributions along elevational gradients [5,6].

In insects, species richness generally declines with increasing elevation. Some studies have linked this pattern to primary productivity [7], whereas others have attributed it to increasing environmental harshness with elevation [6]. Additional work has documented taxa restricted to specific elevational bands, suggesting that distinct environments along a cline may be ecologically irreplaceable [8]. Because insects are integral to ecosystem function, understanding how communities respond to strong abiotic and biotic shifts is essential for anticipating ecological changes driven by climate change [9].

Insects contribute to bioturbation, organic matter decomposition, and the recycling of essential nutrients, thereby sustaining biogeochemical cycles [10,11]. Necrophagous and detritivorous insects accelerate these processes and influence community structure and the flow of energy and matter through trophic networks [12,13]. Despite their ecological and applied relevance, the environmental, temporal, and spatial dynamics of necrophagous and detritivorous insects remain comparatively underexplored [14]. For example, variation in necrophagous communities along altitudinal gradients has received limited attention [14,15]. Among the most relevant necrophagous insects are Calliphoridae (Diptera, Muscomorpha, Oestroidea), a family of approximately 1500 species commonly known as blowflies, bluebottles, or greenbottle flies [16,17]. Calliphorids occur across diverse environments, including native and human-modified habitats such as domestic settings where food and food waste are handled, making them useful models for ecological and forensic studies [16,18].

Here, we evaluated the structure of necrophagous insect communities across sites spanning contrasting elevations in the Mediterranean-type temperate biome of central Chile. This region is a global biodiversity hotspot characterized by high endemism and vulnerability to human-driven disturbances [19]. Central Chile also encompasses the Andean montane elevational cline, where steep elevational change (0 to >2700 masl) occurs over relatively short geographic distances and is accompanied by pronounced shifts in abiotic factors (e.g., UV irradiance and temperature) and biotic factors (e.g., vegetation composition) [20,21].

Under this framework, we characterized the insect species, particularly Calliphoridae (Diptera), associated with cadaveric assemblages and quantified patterns of abundance and richness across localities in central Chile. Finally, we assessed whether any sampled sites harbour specialized taxa that could serve as indicator species with potential value for conservation-oriented research and forensic applications.

## 2. Materials and Methods

### 2.1. Study Sites

**Figure 1:**
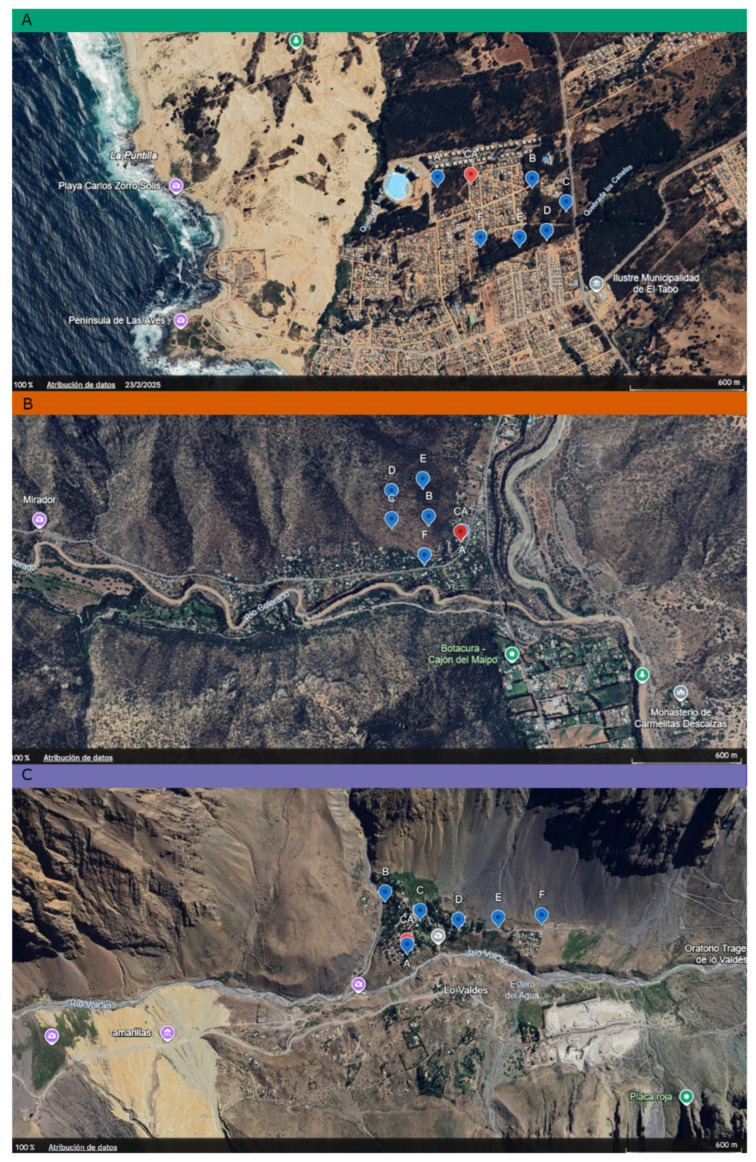
Collected arthropod sites. In (**A**) Las Cruces, El Tabo, Valparaíso region (33°29′12.9″ S: 71°37′33.3″ W), 50 masl, Coastal level; (**B**) Camino al Alfalfal, San José de Maipo, metropolitan region (33°35′37″ S: 70°21′58″ W), 1000 masl, Pre-mountain range level; (**C**) Baños Morales, San José de Maipo, metropolitan region (33°49′28″ S: 70°03′41″ W), 1800 masl, High Andean level. Note that blue markings delimit the coordinates of the collection localities; in the lower right corner are scales of each map generated in Google Earth™.

We selected three sites with distinct environmental profiles and elevation levels, with minimal overlap in vegetation, fauna, and human density (Figure 1):

i. Coastal level: Las Cruces, El Tabo, Valparaíso Region (33°29′12.9″ S: 71°37′33.3″ W), 50 masl. This site is located within the Coastal Scrubland (0 to 700 masl), where the predominant vegetation includes species in the genera *Bahia* (Asteraceae), *Fuchsia* (Onagraceae), *Puya* (Bromeliaceae), *Pouteria* (Sapotaceae), and *Haplopappus* (Asteraceae). The area has a mean annual temperature of 14 °C and mean annual precipitation of 450 mm [22].
ii. Pre-mountain range level: Camino al Alfalfal, San José de Maipo, Metropolitan Region (33°35′37″ S: 70°21′58″ W), 1000 masl. This site is located within the Sclerophyllous Matorral (700 to 1800 masl), where predominant plant species include boldo (*Peumus boldus*), quillay (*Quillaja saponaria*), and litre (*Lithraea caustica*) [20]. Temperatures range from 10 °C to 26 °C, and annual precipitation is approximately 250 mm.
iii. High Andean level: Baños Morales, San José de Maipo, Metropolitan Region (33°49′28″ S: 70°03′41″ W), 1800 masl. This site belongs to the Andean sclerophyllous forest (1650 to 1950 masl) and is dominated by low thickets of *Chuquiraga oppositifolia*, with marginal sclerophyllous forests of *Kageneckia angustifolia* [23]. Annual precipitation is approximately 656 mm, and the mean annual thermal oscillation is 19.2 °C [22].

### 2.2. Insect Collection

We collected organisms using trap-based and active sampling. We used the trap described by Ferreira [24] and later modified by Moretti et al. [25] to incorporate more durable materials, given its recognized efficiency for capturing necrophagous Diptera [26,27,28,29]. Each trap was constructed from two 2 L plastic bottles with the lower third removed and then glued together, leaving the upper section transparent and painting the lower section black [25]. At each site, 12 traps were deployed and checked after 24 h. Pairs of traps with identical bait types were attached to trees at approximately 1.5 m above ground level, whereas pairs with different bait types were placed 200 m apart.

Active sampling was conducted by two collectors at each site during the warmest period of the day (12:00 to 13:30). To attract dipterans, meat was aged for 48 h and stored in plastic containers [29]. Specimens were collected into a plastic bag sealed at the top and then transported in a ventilated box for subsequent identification in the laboratory.

Beef liver and fish were used as baits because they effectively attract necrophagous Diptera [28,29,30]. In both cases, baits were obtained fresh and allowed to decompose at room temperature for 48 h before use.

Sampling was conducted during comparable climatic periods (Table 1). Collections at the Coastal and Pre-mountain sites were performed during the first and second weeks of October 2023, respectively, whereas collections at the High Andean site were performed during the first week of January 2024.

### 2.3. Taxonomic Determination

Field samples were individualized in 1.5 mL tubes and examined under a stereomicroscope at the Laboratory of Sensory Ecology and Plant-Insect Interaction (Entomology Institute, UMCE, Chile) and the Laboratory of Integrative Entomology (UNICAMP, Brazil). Arthropods were initially sorted into higher taxonomic categories (class, order, etc.). Dr. Darko Cotoras (Faculty of Biological Sciences, Pontifical Catholic University of Chile) identified arachnids to family level. A taxonomic key [31] was used to determine Hymenoptera to subfamily level, supplemented by consultation with Dr. Robert R. Kula (Department of Entomology, University of Kansas; United States Department of Agriculture, USA), a specialist in microhymenopteran parasitoids.

Diptera were first identified to family level [32,33] and subsequently to species using taxonomic keys [34,35,36,37,38,39,40,41,42] and comparisons with reference specimens identified by specialists and deposited in the Entomological Collection of the Integrative Entomology Laboratory (CELEI, Brazil) [43].

### 2.4. Analysis of Ecological Parameters

#### 2.4.1. Abundance and Diversity

We used PAST 4.17 [44] to test for differences in insect abundance among sites, first assessing normality. Because abundance data from the High Andean site were not normally distributed (Shapiro–Wilk test: N = 7, W = 0.786, *p* = 0.030), we performed non-parametric comparisons among sites using the Kruskal–Wallis test. When significant differences were detected, pairwise comparisons were performed using a *t*-test.

We calculated richness, evenness, and dominance indices to characterize diversity patterns among taxa directly (necrophagous) and indirectly (predators, parasitoids, and others) associated with the baits. Specifically, we calculated (i) Simpson’s 1 − D (λ), representing the inverse probability that two randomly selected individuals belong to the same taxon; (ii) Shannon–Wiener (H) index, reflecting uncertainty in taxonomic identity such that lower diversity corresponds to higher certainty; and (iii) evenness, quantifying the relative abundance of taxa contributing to richness at a site [45,46].

Species accumulation (rarefaction) curves were generated for each site using the Shannon index (H) as the response variable to evaluate sampling completeness. In addition, Indicator Value (IndVal) scores were compared pairwise among sites with Bonferroni correction.

#### 2.4.2. Community Composition and Indicator Species

To compare community composition among sites, we used the non-parametric ANOSIM (Analysis of Similarities) test [47] implemented in PAST 4.17. We also performed Indicator Species Analysis, which uses permutation testing to identify taxa indicative of the compared groups [48]. This analysis was conducted twice: first using families across all collected taxa to identify indicator taxa, and then using Calliphoridae species only to identify indicator species.

## 3. Results

### 3.1. Sampled Taxa and Climatological Parameters

A total of 1240 arthropods were collected, primarily from the class Insecta, with a single specimen of Arachnida recorded (Table 2). Three insect orders were identified: Diptera (n = 1216), Hymenoptera (n = 22), and Neuroptera (n = 1). All hymenopterans in Braconidae belonged to the subfamily Alysiinae.

Carrion traps yielded 862 arthropods (Coastal, n = 170; Pre-mountain, n = 201; High Andean, n = 491), whereas active sampling in the vicinity of traps yielded 378 additional specimens (Coastal, n = 139; Pre-mountain, n = 111; High Andean, n = 129) (Table 2). All taxa collected by active sampling were also obtained with carrion traps, supporting the hypothesis that these insects are associated with this resource.

**Figure 2:**
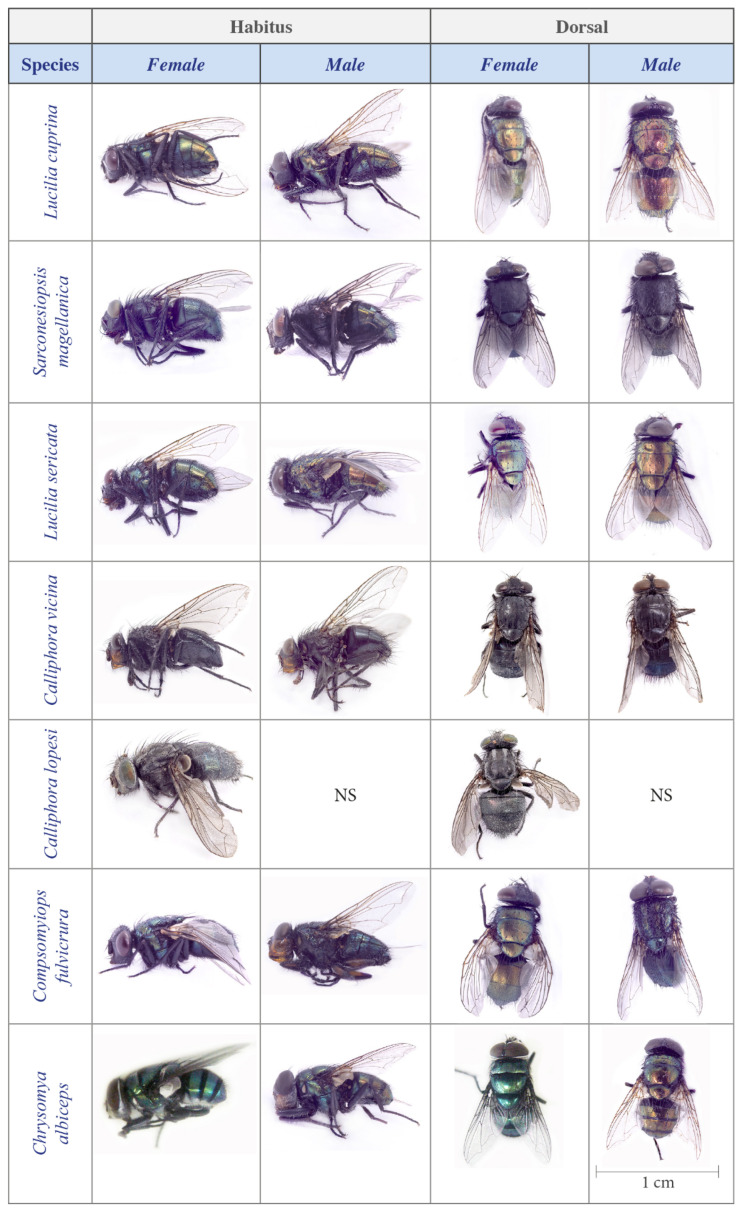
Photographs of male and female representatives of the Calliphoridae species sampled in this study, showing habitus (lateral view) and dorsal view, were taken along distinct sites in Central Chile. Note: scale bar = 1 cm; NS means not sampled.

Within Diptera, families were recorded in descending order of abundance as follows: Calliphoridae (n = 947), Muscidae (n = 118), Piophilidae (n = 77), Fanniidae (n = 44), Sarcophagidae (n = 15), Mycetophilidae (n = 8), Phoridae (n = 5), and Ulidiidae (n = 2). Owing to limitations in available taxonomic keys and taxon-specific constraints (e.g., Sarcophagidae and Fanniidae keys are available only for adult males), species-level identification was performed only for Calliphoridae. The following calliphorid species were identified: *Sarconesiopsis magellanica* (Le Guillou, 1842) (n = 422), *L. cuprina* (Wiedemann, 1830) (n = 303), *Calliphora vicina* Robineau-Desvoidy, 1830 (n = 100), *Lucilia sericata* (Meigen, 1826) (n = 57), *Chrysomya albiceps* (Wiedemann, 1819) (n = 34), *Compsomyiops fulvicrura* (Robineau-Desvoidy, 1830) (n = 27), and *Calliphora lopesi* (Mello, 1962) (n = 4) (Table 3; Figure 2). *Lucilia cuprina*, *L. sericata*, *C. fulvicrura*, *C. albiceps*, and *S. magellanica* were more abundant at the High Andean site, whereas *C. vicina* was more abundant at the Coastal and Pre-mountain sites (Table 3). *C. fulvicrura* was the only species recorded exclusively at a single site (Table 3).

Temperature ranges, maximum temperature, and accumulated precipitation were broadly similar among sites (Table 1). As expected, relative humidity at the Coastal site was approximately twofold higher than at the other sampling sites (Table 1).

### 3.2. Ecological Parameters

#### 3.2.1. Abundance and Diversity

**Figure 3:**
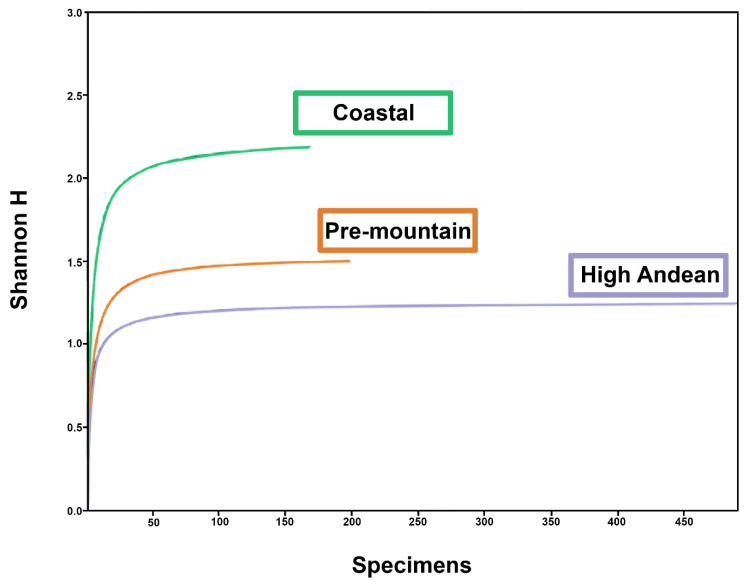
Rarefaction curve of the total specimens collected at each site in Central Chile, represented by the Shannon–Weiner index (H).

**Figure 4:**
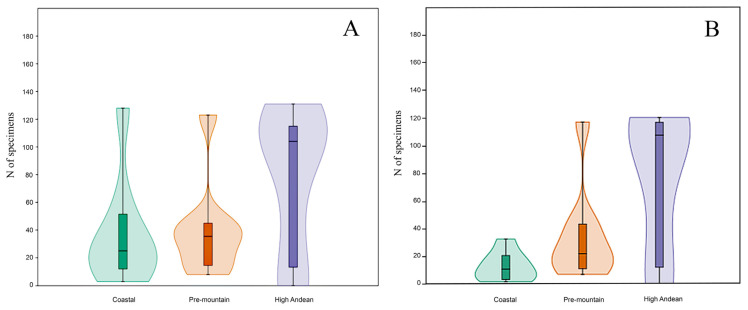
Box plot and violin distribution (mean, medians, and standard error) of the abundances of insects collected in the three sites in Central Chile: (**A**) for all the arthropod taxa; (**B**) for Calliphoridae species.

Based on standardized sampling effort and duration, rarefaction curves indicated adequate sampling depth at the Pre-mountain and High Andean sites, but not at the Coastal site (Figure 3). Total arthropod abundance did not differ significantly among sites (H = 4.842; Hc = 4.964; *p* = 0.0836) (Figure 4). Simpson’s dominance, Shannon–Wiener diversity, and evenness were significantly higher at the Coastal site than at the Pre-mountain (*t* = 6.303; df = 357.12; *p* < 0.001) and High Andean (*t* = 11.69; df = 357.85; *p* < 0.001) sites (Table 2). The Pre-mountain and High Andean sites also differed from each other (*t* = 2.612; df = 326.03; *p* = 0.0094).

**Figure 5:**
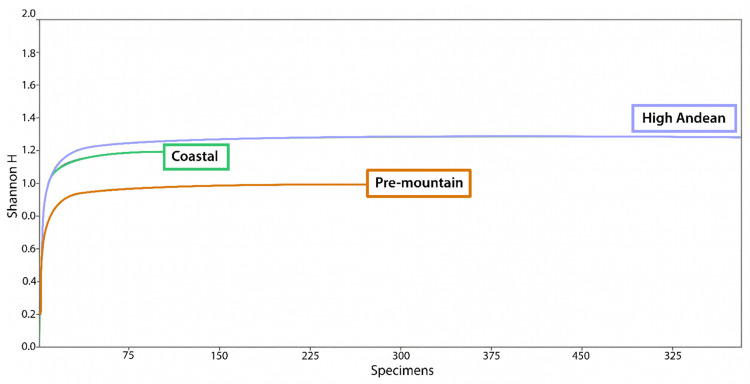
Rarefaction curve of Calliphoridae specimens collected at each site in Central Chile, represented by the Shannon–Weiner index (H).

For Calliphoridae, rarefaction curves reached a plateau at all sites, indicating sufficient sampling to capture representative diversity for this taxon during the study period (Figure 5). Calliphoridae abundance did not differ significantly among sites (H = 3.787; Hc = 3.809; *p* = 0.1489) (Figure 4). Simpson’s dominance, Shannon–Wiener diversity, and evenness values were similar across sites (Table 3) and did not differ significantly by *t*-test (*t* = 1.695; df = 12; *p* = 0.116).

#### 3.2.2. Community Composition and Indicator Values (IndVal)

**Figure 6:**
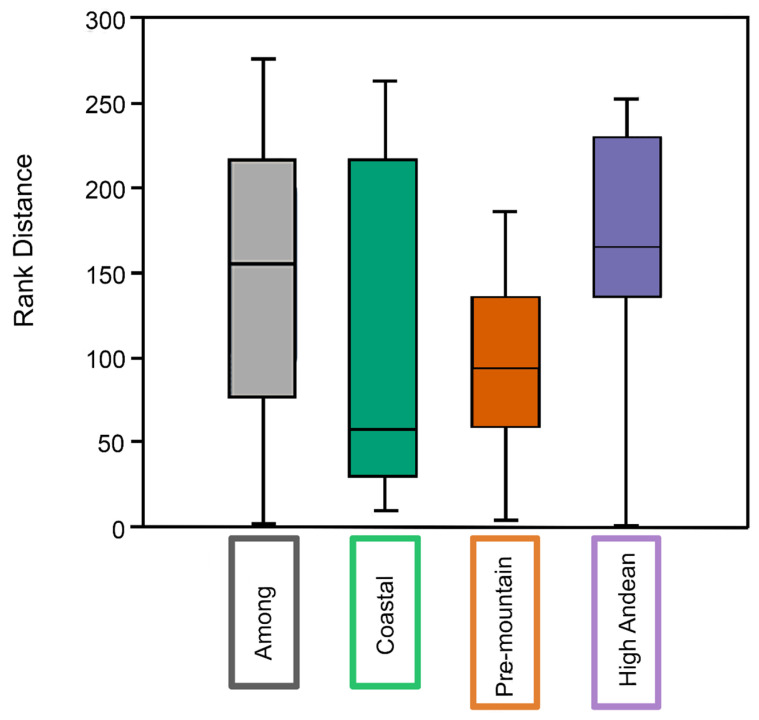
ANOSIM posterior comparison of the distances of the similarity ranges (Y) versus the groups of insects collected with carrion traps in the sampled sites (X). Different letters represent significant differences between groups at *p* = 0.05.

Community composition differed significantly among sites based on ANOSIM (permutations, N = 9999; mean rank within = 87.92; mean rank between = 113; R = 0.239; *p* = 0.003) (Table 2). Post hoc pairwise ANOSIM with Bonferroni correction indicated that the Coastal site differed significantly from the other sites, consistent with Figure 6.

**Figure 7:**
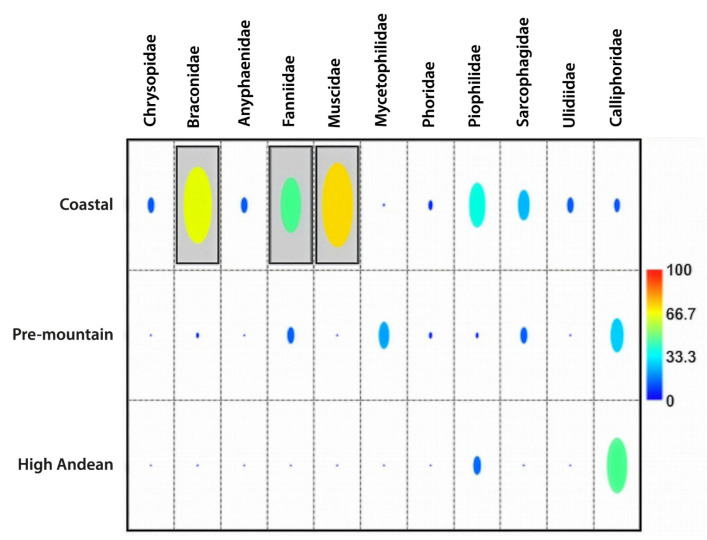
Indicator taxa. The heat plot represents the percentage of Indicator Value (%) of each family, considering its presence at the respective site. The horizontal line shows the taxa names, while the vertical lines show the altitudinal levels considered in this study. The grey background colour indicates that the value is significant and that group qualifies as an indicator.

**Figure 8:**
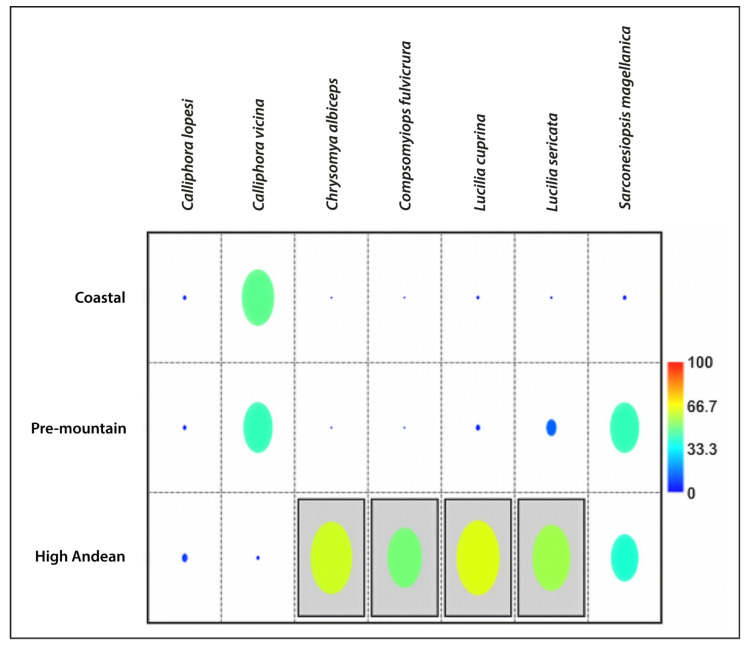
Indicator species. The heat plot represents the percentage of Indicator Value (%) of each Calliphoridae species, considering its presence at the respective site. The horizontal line shows the species names, while the vertical lines show the altitudinal levels considered in this study. The grey background colour indicates that the value is significant and that group qualifies as an indicator.

IndVal analyses identified significant associations at both the arthropod family level and the Calliphoridae species level (Figure 7 and Figure 8; Table 4). At the family level, most indicator taxa were associated with the Coastal site, with Braconidae, Fanniidae, and Muscidae as the strongest indicators (Figure 7). At the species level, the strongest indicators were concentrated at the High Andean site, with *C. albiceps*, *C. fulvicrura*, *L. cuprina*, and *L. sericata* identified as strong indicators of this site (Figure 8). All indicator taxa showed high IndVal values with statistical significance (*p* < 0.05), consistent with strong site associations (Table 4).

## 4. Discussion

Temperature is a primary determinant of insect activity [48]. Accordingly, surveys and diversity assessments often interpret the establishment of local insect populations using seasonal frameworks [14,17,49]. Less frequently, studies classify species by ecological preferences, an approach that can yield important insights into local population dynamics [50,51]. Regardless of approach, substantial knowledge gaps persist regarding fauna associated with habitats across discrete elevational bands, particularly in the Neotropics [52]. The Andes Cordillera, one of the world’s most extensive mountain systems spanning western South America, encompasses pronounced topographic and climatic heterogeneity that can support distinct faunal assemblages. However, documenting biological activity above 1500 m in Chile can be logistically challenging and hazardous (e.g., avalanche risk) and may be inefficient because of reduced biological activity [53,54]. To address these constraints, we surveyed necrophagous insect assemblages across three localities selected for climatic similarity, particularly temperature and precipitation. Notably, even if temperatures are comparable among sites, sampling in different seasons can limit the comparability of insect fauna across an altitudinal gradient because seasonal activity patterns may differ among taxa. Therefore, we recommend that future elevational assessments include additional sites, increased replication, and extended sampling intervals.

Despite a relatively small sample size and a single sampling effort per locality, we recovered meaningful information on the diversity of necrophagous arthropod assemblages in the central temperate zone of Chile, including taxa that are indirectly associated through trophic specialization (e.g., predators and parasitoids). However, consistent with the global taxonomic impediment [29,54], not all specimens could be identified to species level. Consequently, these data are insufficient for a comprehensive evaluation of population dynamics in the region but provide a useful baseline for long-term surveys with broader taxonomic resolution and for assessing impacts associated with anthropogenic or climatic change.

All Calliphoridae (Diptera) species collected here have been reported previously in Chile [35,36,37,38,40,42,55]. Our analyses indicate that necrophagous blowfly composition varies little among the sampled localities. Although *C. fulvicrura* was collected exclusively at the High Andean site, a previous study in Chile reported this species during periods of higher temperature and low precipitation at 14 m elevation [55]. That study also associated *C. fulvicrura* with rural environments near animal breeding sites, whereas our collections were conducted in an urban setting. In a review of Chrysomyinae, Dear [38] examined *C. fulvicrura* specimens collected from 120 to 1930 m across Argentina, Bolivia, Brazil, Chile, and Uruguay, largely from areas distant from urban centres.

Other arthropod taxa and Calliphoridae species identified as indicators showed strong associations with their respective sampling localities. This pattern underscores the local ecological relevance of these taxa, particularly at higher elevations, where abiotic constraints can reduce survival of multicellular organisms [1,20,56]. Carrion flies exploit ephemeral resources [32], and their activity contributes to organic matter recycling, complementing microbial decomposition and supporting soil formation and recovery [10,11,13]. Carrion-associated insects also provide additional ecosystem services. For example, some Calliphoridae participate in outcrossing pollination, particularly of plant species with floral odours resembling decaying meat [57]. We also recorded higher-trophic-level taxa directly associated with Calliphoridae. Alysiinae (Hymenoptera: Braconidae), an indicator taxon of the Coastal site, are endoparasitoids of second- and third-instar dipteran larvae [58,59]. Given the trophic role of Calliphoridae, identifying the abiotic drivers or disturbances that reduce their abundance is important because declines may indicate environmental degradation [17].

Beyond ecological and conservation relevance, our findings may inform forensic investigations, particularly in regions characterized by mountainous ecosystems with steep elevational gradients [60]. When human remains are recovered at a given elevational band, entomological evidence can be collected and evaluated. The absence of taxa expected for that elevation, or the presence of taxa typical of another locality, may suggest post-mortem body movement and potential concealment of homicide [61]. Indicator taxa may also help infer the likely location of death. All Calliphoridae species collected here have been reported from decomposing human remains and have associated biological and ecological data used to estimate post-mortem interval in forensic contexts, with the exception of the necrophagous *C. fulvicrura* [54,61]. To support routine application, additional faunal inventories are needed, ideally integrated with case records documenting recovery locations, to validate the reliability of the indicator taxa proposed here.

In summary, this study provides baseline information on necrophagous arthropod assemblages, particularly Diptera, in central Chile and supports further investigation of these taxa in ecological, conservation, and forensic entomology contexts. These data should facilitate regional characterization of saprophagous entomofauna, identification of indicator species, and evaluation of how climate change may reshape community diversity. In addition, population genetic analyses of key species, including *Lucilia cuprina*, *L. sericata*, *C. albiceps*, and *C. fulvicrura*, would help clarify patterns of structure and connectivity across elevational gradients.

## 5. Conclusions

Necrophagous arthropod assemblages differed in abundance and community composition among localities in central Chile. The Coastal site (Las Cruces) exhibited the highest diversity, whereas the High Andean site (Baños Morales) showed the lowest. These patterns suggest that abiotic environmental factors strongly influence the distribution of some taxa. However, no significant differences were detected among localities in the richness or composition of carrion-associated Diptera, particularly Calliphoridae.

At the Coastal site, Braconidae (Hymenoptera) and the dipteran families Fanniidae and Muscidae were identified as significant indicator taxa. At the High Andean site, the calliphorid species *Chrysomya albiceps*, *Compsomyiops fulvicrura*, *Lucilia cuprina*, and *Lucilia sericata* were significant indicators.

All Calliphoridae species collected in this study have previously been reported to develop on decomposing human remains in the Neotropics, with the exception of *C. fulvicrura*. Overall, these findings underscore the importance of systematic faunal surveys of necrophagous insects across environmental gradients, including seasonality and altitude, to develop robust baseline datasets that support forensic practice.

## Figures and Tables

**Table 1:** Minimum, maximum, and average ambient temperature (°C), relative air humidity (%), and accumulated precipitation (mm) measured locally during arthropod sampling.

Sites	Temperature (°C)			Humidity (%)			Precipitation (mm)
	Average	Minimum	Maximum	Average	Minimum	Maximum	
Coastal	16.5	14.5	22.4	62.5	51.5	72.5	0
Pre-mountain	24.7	21.1	28.9	30.0	27.0	36.5	0
High Andean	25.5	23.0	28.8	26.0	23.5	27.5	0

**Table 2:** Carrion trapped and actively sampled abundance and ecological parameters of arthropods, by family and by site.

Class	Order	Family/ Subfamily	Sites		
			Coastal	Pre-Mountain	High Andean
Arachnida	Araneae	Anyphaenidae	1	0	0
Insecta	Neuroptera	Chrysopidae	1	0	0
	Hymenoptera	Braconidae	19	3	0
	Diptera	Ulidiidae	2	0	0
		Sarcophagidae	6	8	1
		Piophilidae	38	8	31
		Phoridae	3	2	0
		Mycetophilidae	1	7	0
		Muscidae	111	2	5
		Fanniidae	27	16	1
		Calliphoridae	99	266	582
Abundance			308	312	620
Taxa number			11	8	5
Simpson’s Dominance Index (1 − D)			0.742	0.269	0.116
Shannon–Weiner Index (H)			1.603	0.682	0.272
Evenness (e^H/S^)			0.451	0.247	0.263

**Table 3:** Carrion trapped and actively sampled abundance and ecological parameters of Calliphoridae species by site.

Species	Sites		
	Coastal	Pre-Mountain	High Andean
*Calliphora lopesi* (Mello)	1	1	2
*Calliphora vicina* (Robineau-Desvoidy)	47	48	5
*Lucilia cuprina* (Wiedemann)	23	27	253
*Lucilia sericata* (Meigen)	3	12	42
*Compsomyiops fulvicrura* (Robineau-Desvoidy)	0	0	27
*Chrysomya albiceps* (Wiedemann)	0	1	33
*Sarconesiopsis magellanica* (Le Guillou)	25	177	220
Abundance	99	266	582
Taxa number	5	6	7
Simpson’s Dominance Index (1 − D)	0.663	0.514	0.659
Shannon–Weiner Index (H)	1.21	1.00	1.29
Evenness (e^H/S^)	0.673	0.455	0.519

**Table 4:** Main taxa and species indicators, according to IndVal (%) and *p* values for each site.

Sites	Indicator Taxa	IndVal (%)	*p*-Value	Indicator Species	IndVal (%)	*p*-Value
Coastal	Braconidae	64.29	0.0017	-	-	-
	Fanniidae	46.02	0.0193			
	Muscidae	70.59	0.0026			
Pre-mountain	-	-	-	-	-	-
High Andean	-	-	-	*Chrysomya albiceps* (Wiedemann)	60.66	0.0021
				*Compsomyiops fulvicrura* (Robineau-Desvoidy)	50.00	0.0058
				*Lucilia cuprina* (Wiedemann)	62.62	0.0036
				*Lucilia sericata* (Meigen)	55.26	0.0112

## Data Availability

The original contributions presented in this study are included in the article. Further inquiries can be directed to the corresponding authors.
